# Dual dynamics of mitochondrial permeability transition pore opening

**DOI:** 10.1038/s41598-020-60177-1

**Published:** 2020-03-03

**Authors:** Benjamin Wacquier, Laurent Combettes, Geneviève Dupont

**Affiliations:** 1 Unit of Theoretical Chronobiology, Faculté des Sciences, Université Libre de Bruxelles (ULB) CP231, B1050 Brussels, Belgium; 2 UMR-S 1174, Université Paris-Sud, INSERM, 91405 Orsay, France

**Keywords:** Calcium signalling, Computational models

## Abstract

Mitochondria play an essential role in bioenergetics and cellular Ca${}^{2+}$ handling. The mitochondrial permeability transition pore (mPTP) is a non-specific channel located in the inner mitochondrial membrane. Long-lasting openings of the pore allow the rapid passage of ions and large molecules, which can result in cell death. The mPTP also exhibits transient, low conductance openings that contribute to Ca${}^{2+}$ homeostasis. Although many regulators of the pore have been identified, none of them uniquely governs the passage between the two operating modes, which thus probably relies on a still unidentified network of interactions. By developing a core computational model for mPTP opening under the control of mitochondrial voltage and Ca${}^{2+}$, we uncovered the existence of a positive feedback loop leading to bistability. The characteristics of the two stable steady-states correspond to those of the two opening states. When inserted in a full model of Ca${}^{2+}$ handling by mitochondria, our description of the pore reproduces observations in mitochondrial suspensions. Moreover, the model predicted the occurrence of hysteresis in the switching between the two modes, upon addition and removal of free Ca${}^{2+}$ in the extra-mitochondrial medium. Stochastic simulations then confirmed that the pore can undergo transient openings resembling those observed in intact cells.

## Introduction

In most cell types, cytosolic Ca${}^{2+}$ is a key ion that controls major intracellular processes in health and disease^1^. The signalling specificity of this ion largely relies on the spatio-temporal organisation of its stimulus-induced increases^2^. For example, oscillations and waves can occur due to the auto-catalytic Ca${}^{2+}$ release from the endoplasmic reticulum (ER), which acts as the main Ca${}^{2+}$ store. However, Ca${}^{2+}$ exchanges with other organelles further extend the spatio-temporal diversity of Ca${}^{2+}$ signals. Among these, exchanges between the cytosol and mitochondria play an important role. Entry of Ca${}^{2+}$ into mitochondria is mediated by the mitochondrial Ca${}^{2+}$ uniporter (MCU), while exit occurs via the Na${}^{+}$-Ca${}^{2+}$ and the H${}^{+}$-Ca${}^{2+}$ exchangers (NCLX and HCX), in non-excitable cells^3^. Ca${}^{2+}$ uptake by mitochondria not only participates in the regulation of the cytosolic Ca${}^{2+}$ concentration ([Ca${}^{2+}$]) but also stimulates mitochondrial respiration and ATP production^4^.

The mitochondrial permeability transition pore (mPTP) can also transport Ca${}^{2+}$. Indeed, in response to a metabolic stress or to an excessive accumulation of mitochondrial Ca${}^{2+}$, an increase in the permeability of the inner mitochondrial membrane (IMM) can be observed. First described in the 1970’s^5–7^, this phenomenon, called permeability transition, could rapidly be ascribed to a non-selective pore. Mitochondrial Ca${}^{2+}$ overload and/or oxidative stress lead to a massive and unselective opening of the pore, which allows for the transit of molecules up to 1500 Da. This includes Ca${}^{2+}$, metabolic substrates and ATP. Consequently, opening of the mPTP in this mode induces the dissipation of the IMM voltage ($\Delta \Psi$) and, finally, cellular death. However, the pore can also exhibit moderate and transient openings^8^. Indeed, the existence of smaller conductance sub-states of the mPTP has been demonstrated by experiments on mitochondrial suspensions^9^ and by electrophysiology^10,11^. This mode of reduced activity contributes to Ca${}^{2+}$ homeostasis and thereby helps maintaining normal cellular functions^8,12^.

**Figure 1:**
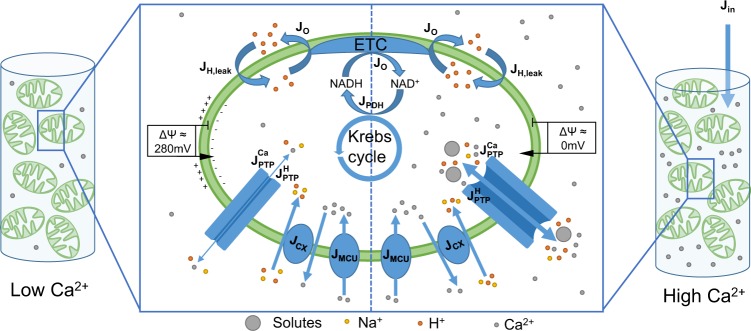
Schematic representation of the model describing Ca${}^{2+}$ dynamics and mPTP opening in mitochondrial suspensions. The model includes Ca${}^{2+}$ exchanges between mitochondria and the extra-mitochondrial medium through the Mitochondrial Ca${}^{2+}$ Uniporter (${J}_{MCU}$), the Ca${}^{2+}$ exchangers (${J}_{CX}$), and the mPTP (${J}_{PTP}^{Ca}$). The mPTP is potentially permeable to ions (H${}^{+}$, Mg${}^{2+}$, K${}^{+}$, ...) and small solutes. These fluxes are gathered in ${J}_{PTP}^{H}$. Mitochondrial Ca${}^{2+}$ triggers the reduction of NAD${}^{+}$ in NADH (${J}_{PDH}$) that can be oxidised by the electron transport chain (${J}_{O}$) to generate a proton gradient. A leakage of protons into the mitochondrial space is considered (${J}_{H,leak}$). ATP synthesis is not included because the medium does not contain adenine nucleotides. The model reproduces a resting state (on the left) and a Ca${}^{2+}$-overloaded state (on the right). At rest, the Ca${}^{2+}$ concentration in the suspension is low. Thus, the mPTP does not open fully, and the fluxes through this pore (${J}_{PTP}^{H}$, ${J}_{PTP}^{Ca}$) are low. In these conditions, a $\Delta \Psi$ is maintained thanks to the extrusion of protons. Upon the addition of a massive amount of Ca${}^{2+}$ in the medium (${J}_{in}$), the mPTP opens and becomes highly permeable to ions and solutes. This leads to the dissipation of the $\Delta \Psi$. See text, SI Appendix and Wacquier *et al*.^22^ for more details on the model.

Well before its plausible molecular identification^13,14^, it was known that many factors, including reactive oxygen species (ROS), pH, inorganic phosphates or cyclophilin D regulate the permeability transition^12^. Importantly, the main drivers of mPTP opening are $\Delta \Psi$ and mitochondrial Ca${}^{2+}$ concentration ([Ca${}^{2+}$]${}_{{\rm{m}}}$). Opening requires a low voltage and a high Ca${}^{2+}$ load. Once open, the mPTP allows for the passage of ions, including Ca${}^{2+}$ itself, which leads to mitochondrial membrane depolarisation (Fig. 1). There is no evidence of any specific regulator that would drive the mPTP from a low- to a large opening state, and it can thus be anticipated that this passage results from a network of feedback regulations.

In this study, we hypothesised that the two operating states of the mPTP rely on bistability. In such a scenario, two stable steady-states can coexist in a range of [Ca${}^{2+}$]${}_{{\rm{m}}}$, which confers robustness to both states of channel opening. This assumption is based on the observation of a positive feedback loop that relies on the cross-inhibition between mPTP opening and mitochondrial voltage ($\Delta \Psi$): low voltage indeed opens the mPTP, which leads to further mitochondrial depolarisation (i.e. decrease in $\Delta \Psi$). As mitochondrial Ca${}^{2+}$, known as the main mPTP regulator, directly controls $\Delta \Psi$, we anticipated that this positive feedback loop constitutes the core regulatory mechanism of mPTP opening. We first built a computational model formalising the mechanism just described. As assumed, the model can display bistability. We validated the model and parameter values using experiments in mitochondrial suspensions, which allow for controlled Ca${}^{2+}$ exchanges between mitochondria and the extra-mitochondrial medium (em). The model then led us to rightly predict the conditions in which a hysteretic behaviour, which is a hallmark of bistability, can be observed. The robustness of bistability was assessed by a sensitivity analysis of the computational model. Finally, we showed that the model recapitulates the reversible, transient openings of the mPTP observed in intact cells. In conclusion, this work proposes a simple dynamical mechanism by which mitochondria can safely use the mPTP in two operating modes, which differ drastically by their conductances and by their physiological implications. Put in a cellular context, the proposed bistable behaviour not only provides robustness to the Ca${}^{2+}$ transport properties of the mPTP, but also ensures that, once initiated, the mPTP-induced mitochondrial depolarisation is physiologically irreversible.

## Model

**Figure 2:**
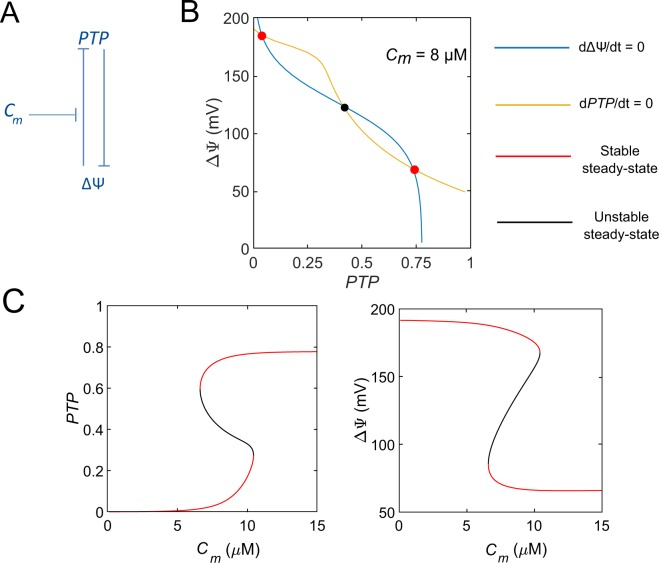
A two-variable model describing mPTP opening. (**A**) Scheme of the model. The positive feedback loop between the fraction of open mPTP ($$PTP$$) and mitochondrial voltage ($$\Delta \Psi $$) underlies bistability. This loop results from cross-inhibition. $${C}_{m}$$ modulates the threshold of $$PTP$$ inhibition by $$\Delta \Psi $$. (**B**) Phase space for $${C}_{m}=8$$$$\mu M$$. The yellow and blue curves represent $$\Delta \Psi $$ and $$PTP$$ null-clines, respectively. $${C}_{em}$$ was fixed at 0.5 $$\mu M$$ and $$NADH$$ at 200 $$\mu M$$. In red: stable steady-state. In black : unstable steady-state. (**C**) Bifurcation diagrams of $$PTP$$ and $$\Delta \Psi $$ as a function of $${C}_{m}$$.

The regulation of the mPTP by [Ca${}^{2+}$]${}_{{\rm{m}}}$ and $\Delta \Psi$ is schematised in Fig. 2A. Based on this scheme, a single differential equation is used to describe the evolution of the fraction of open mPTP in the mitochondrial pool, noted $PTP$. A small value of $PTP$ thus corresponds to the transient, low conductance mode while a high value of this variable can be associated to the large, long-lasting opening mode. In the following, we refer to these states as the low and high conductance modes. It should be noted that these terms do not refer to the single channel activities measured by electrophysiology. As in previous models^15,16^, the evolution equation includes a highly non-linear term of opening of the mPTP, that is triggered when $\Delta \Psi$ falls below a threshold^17,18^. In agreement with experimental data^19,20^, the value of the threshold is controlled by [Ca${}^{2+}$]${}_{{\rm{m}}}$ (${C}_{m}$ in the model). The rate of mPTP closure is described by a linear function. Thus, the evolution of the fraction of open mPTP is given by :

1 $$\frac{dPTP}{dt}={V}_{op}(1-PTP)\frac{1}{1+{e}^{\frac{\Delta \Psi -{q}_{op}\cdot {C}_{m}}{{q}_{11}}}}-{k}_{cl}\cdot PTP,$$

where ${V}_{op}$ and ${k}_{cl}$ are rate constants of mPTP opening and closing, respectively. ${q}_{op}$ and ${q}_{11}$ set the Ca${}^{2+}$ and the voltage dependencies of mPTP opening. When the mPTP is open, Ca${}^{2+}$ and protons leak through the pore. Each ion flux depends on the electrochemical gradient and on the opening state of the mPTP. We describe these fluxes by mathematical expressions based on a simplified version of the Goldman-Hodgkin-Katz formalism^21^. Fluxes of Ca${}^{2+}$ and H${}^{+}$ are described by Eqs. 2 and 3, respectively.

2 $${J}_{PTP}^{Ca}={V}_{PTP}^{Ca}\cdot PTP\cdot \frac{({C}_{m}-{C}_{em})}{1+{e}^{\frac{{q}_{13}-\Delta \Psi }{{q}_{12}}}},$$

3 $${J}_{PTP}^{H}={V}_{PTP}^{H}\cdot PTP\cdot \frac{1}{1+{e}^{\frac{{q}_{13}-\Delta \Psi }{{q}_{12}}}},$$

where ${V}_{PTP}^{Ca}$ and ${V}_{PTP}^{H}$ are rate constants of Ca${}^{2+}$ and H${}^{+}$ fluxes through the mPTP, and ${q}_{13}$ and ${q}_{12}$ are coefficients characterising the voltage dependencies of these ions fluxes through the mPTP. The concentration of H${}^{+}$ does not appear in Eq. 3 as it is not a variable of the model. It is therefore implicitly included in ${V}_{PTP}^{H}$, and the flux ${J}_{PTP}^{H}$ only appears in the evolution equation for voltage (Eq. 4). Moreover, ${J}_{PTP}^{H}$ also includes other compounds leaking through the pore (ions and small solutes).

Experimentally, the dynamics of mPTP opening are commonly investigated in suspensions of mitochondria. To model this experimental system, Eqs. 1–3 describing mPTP opening and closing, and the associated ion fluxes, are included in a previously-published model describing Ca${}^{2+}$ dynamics in such a system (Fig. 1)^22^. This model describes 1) Ca${}^{2+}$ exchanges between mitochondria and their medium via the MCU (${J}_{MCU}$) and the NCLX/HCX (${J}_{CX}$) 2) The Ca${}^{2+}$-dependent synthesis of NADH by the Krebs cycle (${J}_{PDH}$), and its consumption by the electron transport chain (ETC, ${J}_{O}$) 3) The variations of voltage induced by Ca${}^{2+}$ transporters, the ETC and proton leaks (${J}_{H,leak}$). Eq. 1 is thus coupled with this model.

The mitochondrial voltage varies in time according to :

4 $$\frac{d\Delta \Psi }{dt}=({a}_{1}.{J}_{O}-{J}_{H,leak}-{J}_{CX}-2.{J}_{MCU}+2.{J}_{PTP}^{Ca}-{J}_{PTP}^{H})/{C}_{p}$$

Positive voltage corresponds to an excess of positive charges in the extra-mitochondrial medium. ${C}_{p}$ scales molecular fluxes into voltage changes. It includes the membrane capacitance and the Faraday constant. ${a}_{1}$ scales NADH consumption into voltage variations.

For the time evolution of extra-mitochondrial Ca${}^{2+}$ concentration (${C}_{em}$), we write

5 $$\frac{d{C}_{em}}{dt}={f}_{em}({J}_{in}+\delta {J}_{CX}-\delta {J}_{MCU}+\delta {J}_{PTP}^{Ca}),$$

where ${f}_{em}$ is the Ca${}^{2+}$ buffering capacity of the medium considering the rapid buffering approximation^23^. It corresponds to the ratio between free and total Ca${}^{2+}$ concentrations in the extra-mitochondrial medium (em). $\delta$ is the volumic ratio between mitochondria and their medium (${V}_{m}/{V}_{em}$). ${J}_{in}$ allows to simulate the addition of Ca${}^{2+}$ in the medium.

In a similar way, the time evolution of mitochondrial Ca${}^{2+}$ concentration (${C}_{m}$) is expressed by

6 $$\frac{d{C}_{m}}{dt}={f}_{m}({J}_{MCU}-{J}_{CX}-{J}_{PTP}^{Ca}),$$

where ${f}_{m}$ is the Ca${}^{2+}$ buffering capacity of mitochondria.

Finally, NADH is produced by the Krebs cycle and consumed in the ETC, which is described by

7 $$\frac{d[NADH]}{dt}={J}_{PDH}-{J}_{O}$$

The detailed kinetic expressions of the fluxes are exposed in the Supplementary Material. Parameters values are listed in Table S1 and, except for the mPTP opening description, are taken from our previous models that were validated against experimental data^22,24^.

## Results

### The mPTP acts as a bistable switch

Bistability often occurs in a system including a positive feedback loop^25,26^. As schematised in Fig. 2A, such a loop exists between the mPTP and $\Delta \Psi$, as a high $\Delta \Psi$ prevents the opening of the pore, while $\Delta \Psi$ is dissipated by an open mPTP due to ion leakage^8^. Because [Ca${}^{2+}$]${}_{{\rm{m}}}$ controls the value at which $\Delta \Psi$ starts inhibiting mPTP opening, Ca${}^{2+}$ could play a key role in the switch. To study the possible existence of a Ca${}^{2+}$-controlled bistability in the mathematical description of the mPTP presented in the previous section, we first considered a minimal model that only considers the evolution of $\Delta \Psi$ and $PTP$ (Eqs. 1 and 4) while ${C}_{m}$, ${C}_{em}$ and $NADH$ are kept constant. We analysed the behaviour of this two-variable model on the basis of bifurcation diagrams where the steady-states of $\Delta \Psi$ and $PTP$ are shown as a function of ${C}_{m}$ (Fig. 2C). For low ${C}_{m}$ ($<$7 $\mu M$), the system tends towards a resting state, i.e. a large $\Delta \Psi$ and an almost closed mPTP (low value of variable $PTP$). For high ${C}_{m}$ ($>$11 $\mu M$), the system will always evolve towards a dissipated potential and a fully open mPTP. For intermediate concentrations, one can observe the coexistence between these two stable steady-states, separated by an unstable one. This scenario is also visible in the phase plane, where the null-clines intersect once at low or high value of $PTP$, for low or high ${C}_{m}$ respectively (not shown), and three times for intermediate Ca${}^{2+}$ concentrations (Fig. 2B). The two steady-states display the characteristics of the two operating modes of the mPTP described above. The first one occurs at low Ca${}^{2+}$, when a high $\Delta \Psi$ is established across the IMM^8^. The other state is reminiscent of the behaviour of the mPTP reported at high mitochondrial Ca${}^{2+}$ load: the mPTP largely opens, leading to the dissipation of $\Delta \Psi$. Interestingly, in suspensions, it is possible to come back from the high to the low conductance mode by adding a Ca${}^{2+}$ chelator^27^. The model is possibly in agreement with this reversible bistability, as the ordinate axis does not intersect the bistable area^26^.

**Figure 3:**
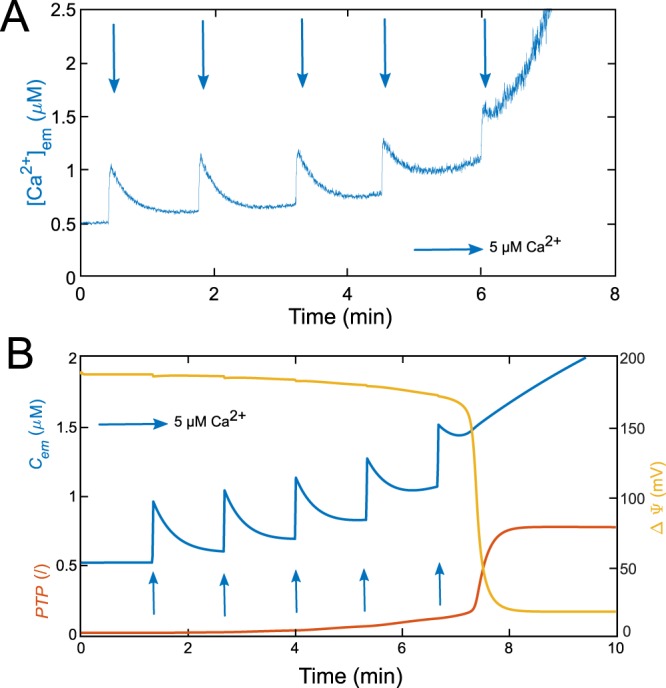
Successive additions of Ca${}^{2+}$ leading to mPTP opening in experiments and in the model. (**A**) Experiment performed in a suspension of mitochondria extracted from rat hepatocytes. The [Ca${}^{2+}$]${}_{{\rm{e}}{\rm{m}}}$ is monitored by fluorescence using 5 $\mu M$ Fluo-4. Following the addition of five Ca${}^{2+}$ pulses (blue arrows), the mPTP opens, as attested by the sudden rise in Ca${}^{2+}$ concentration in the medium. (**B**) Simulations of the five-variable model (Eqs. 1 and 4–7). The model successfully reproduces the [Ca${}^{2+}$]${}_{{\rm{e}}{\rm{m}}}$ behaviour (in blue), in response to the Ca${}^{2+}$ pulses (implemented with ${J}_{in}$). The final increase in the ${C}_{em}$ is associated with a sudden increase in mPTP opening (variable $PTP$, standing for the fraction of open PTP, in red) and a fall in $\Delta \Psi$ (in yellow). See Table S1 for parameter values.

To validate the model, its behaviour must be compared to experiments. Successive additions of large amounts of Ca${}^{2+}$ in a suspension of mitochondria isolated from hepatocytes can induce mPTP opening. In Fig. 3A, each arrow represents the addition of 5 $\mu$M exogenous Ca${}^{2+}$. Changes in Ca${}^{2+}$ concentration in the medium ([Ca${}^{2+}$]${}_{{\rm{e}}{\rm{m}}}$) are monitored by spectrophotometry^22^, allowing to follow Ca${}^{2+}$ exchanges between mitochondria and the medium. Up to the first four additions of Ca${}^{2+}$ in the medium, most of the Ca${}^{2+}$ is sequestered by mitochondria via the MCU, and the mPTP has a limited effect^22^. However, after the fifth addition, one observes a brutal increase in the [Ca${}^{2+}$]${}_{{\rm{e}}{\rm{m}}}$ (Fig. 3A). This important rise is attributed to the opening of the mPTP in its high conductance mode. This opening is accompanied by the release of Ca${}^{2+}$ from mitochondria, which explains the fast and sudden elevation in the medium. To compare the behaviour of the model with these experimental observations, we took variations of Ca${}^{2+}$ and NADH concentrations into account and simulated the whole dynamical system, defined by Eqs. 1 and 4–7 with the same values of parameters as in our previous study^22^. The model faithfully reproduces the experimentally observed behaviour (Fig. 3B): after a few pulses, ${C}_{em}$ increases all of a sudden (blue curve). This increase is associated with the opening of the mPTP (red curve), which triggers a Ca${}^{2+}$ flux from mitochondria to the medium (via ${J}_{PTP}^{Ca}$) while $\Delta \Psi$ is collapsing due to the important ion fluxes through the mPTP (yellow curve).

**Figure 4:**
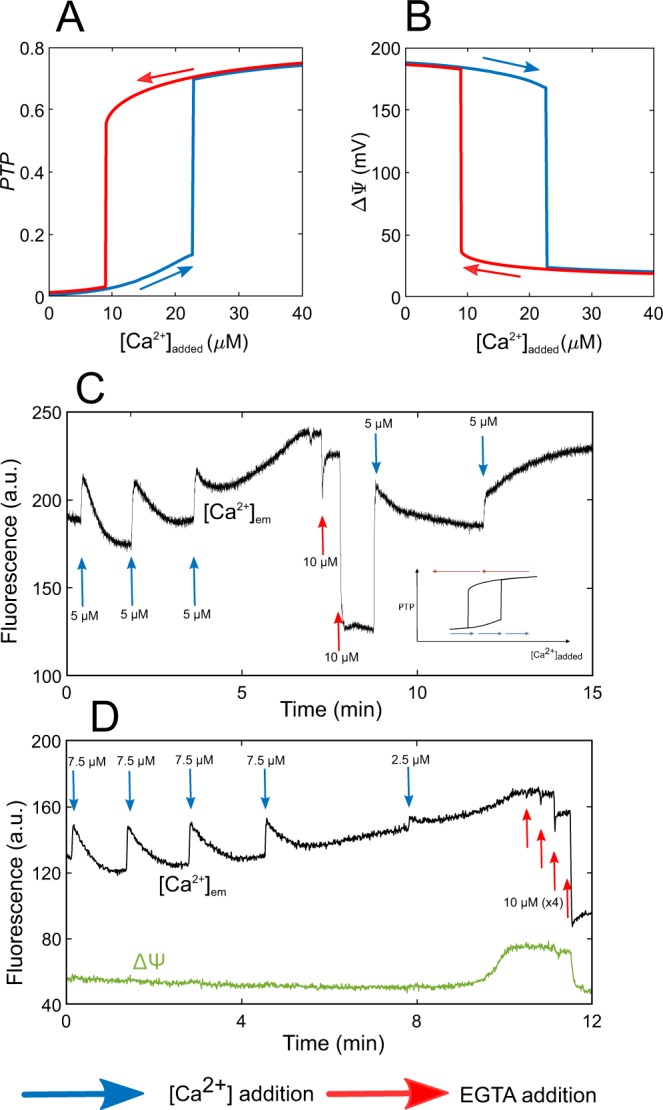
mPTP opening corresponds to a bistable switch. Numerical bifurcation diagrams of the fraction of open mPTP, $$PTP$$ (**A**) and $$\Delta \Psi $$ (**B**) as a function of added Ca$${}^{2+}$$. Blue and red curves stand for trajectories following Ca$${}^{2+}$$ additions (from left to right) or Ca$${}^{2+}$$ removal (from right to left), respectively. The equivalent bifurcation diagrams of $${C}_{m}$$ and $${C}_{em}$$ as a function of added Ca$${}^{2+}$$ are shown in Fig. S1. (**C,D**) Experimental evidence for bistability of the mPTP in a hepatocyte mitochondrial suspension. Ca$${}^{2+}$$ is monitored in the medium with 5 $$\mu $$$$M$$ Fluo-4 (**C**,**D**) and $$\Delta \Psi $$ with 5 $$\mu $$$$M$$ TMRM (SI Appendix) (**D**). In both cases, the concentration of EGTA necessary to close the mPTP is larger than the concentration of Ca$${}^{2+}$$ that allowed its opening. This highlights the existence of hysteresis as schematised in the inset in Panel (C). Panels (C,D) are representative of 12 similar experiments.

The switch-like behaviour shown in Fig. 3 is compatible with the existence of bistability, but might also rely on the existence of a sharp threshold. To investigate if the switch corresponds to a change of steady-state, we cannot draw bifurcation diagrams as a function of ${C}_{m}$ as in the two-variable model (Fig. 2C), since ${C}_{m}$ is a variable in the full model. We thus established pseudo bifurcation diagrams showing the values of $PTP$ and $\Delta \Psi$ as a function of the amount of Ca${}^{2+}$ added during the pulses (Figs. 4A,B and S1). The blue curve is drawn by performing a series of simulations where the steady-states of the model are sequentially computed after successive additions of 0.1 $\mu M$ Ca${}^{2+}$. After a critical amount of added Ca${}^{2+}$, the system is driven towards a state with a large opening of the mPTP. This opening is, as expected, associated with a drop in $\Delta \Psi$ and Ca${}^{2+}$ efflux from mitochondria. From this state, we then simulated the removal of Ca${}^{2+}$ from the medium (red curve corresponding to successive decreases of Ca${}^{2+}$ of 0.1 $\mu$M). Biologically, it would correspond to the addition of a Ca${}^{2+}$ chelator. It is clear that the blue and red trajectories do not coincide and that bistability also occurs in the full model. The amount of Ca${}^{2+}$ that has to be removed to bring the mPTP back in its low conductance mode exceeds the amount of Ca${}^{2+}$ that was necessary to trigger the passage from the low to the high conductance mode. This hysteretic behaviour is typical of a bistable system^26^.

### Hysteresis in mPTP opening

We next wondered if experiments can validate the hysteresis predicted by the model. We first checked in a control experiment that in a medium devoid of mitochondria, the addition of a given amount of Ca${}^{2+}$ is rapidly counterbalanced by the addition of the same amount of the high affinity Ca${}^{2+}$ buffer EGTA (Fig. S2). This observation is in agreement with the 1 to 1 stoichiometry of Ca${}^{2+}$ binding to EGTA and with the values of ${k}_{on}$ and ${k}_{off}$ (6 $\mu$M${}^{-1}$ s${}^{-1}$ and 1 s${}^{-1}$, respectively^28^). Then, on the basis of the model prediction shown in Fig. 4, we submitted mitochondria in suspensions to successive additions of Ca${}^{2+}$, up to the opening of the mPTP. As soon as the mPTP opens, we rapidly added EGTA. We then compared the amount of EGTA necessary to close the mPTP to the amount of Ca${}^{2+}$ that was necessary to open it. Following the addition of three Ca${}^{2+}$ pulses of 5 $\mu$M in a mitochondrial suspension, the mPTP opens and [Ca${}^{2+}$]${}_{{\rm{e}}{\rm{m}}}$ starts to rise (Fig. 4C). Thus, between 10 and 15 $\mu$M Ca${}^{2+}$ were necessary to trigger opening. Accordingly, in the absence of hysteresis, the removal of 5 $\mu M$ Ca${}^{2+}$ should close the mPTP. Nevertheless, the addition of 10 $\mu$M EGTA is not sufficient to close the mPTP: the [Ca${}^{2+}$]${}_{{\rm{e}}{\rm{m}}}$ is still high, indicating that the mPTP is still open. By contrast, the Ca${}^{2+}$ level decreases drastically after a second addition of 10 $\mu$M EGTA. At this stage, the mPTP is closed or in a low conducting mode. As a control, upon Ca${}^{2+}$ re-addition, we see a characteristic spike due to Ca${}^{2+}$ entry in mitochondria. This would not be visible with an open mPTP, as mitochondria would not be able to sequester Ca${}^{2+}$. Finally, a further Ca${}^{2+}$ addition allows to open the mPTP again.

The existence of hysteresis is also supported by the simultaneous measurement of $\Delta \Psi$ and [Ca${}^{2+}$]${}_{{\rm{e}}{\rm{m}}}$ during a similar experiment (Fig. 4D). After 32.5 $\mu M$ of added Ca${}^{2+}$, the drop in potential associated to mPTP opening is observed through an increase in the fluorescence of the probe. Concomitantly, the opening of the pore is seen via the increase in the [Ca${}^{2+}$]${}_{{\rm{e}}{\rm{m}}}$. 40 $\mu M$ EGTA are then required to bring the system back to its basal state, in agreement with the presence of a hysteresis loop.

### Robustness of bistability

**Figure 5:**
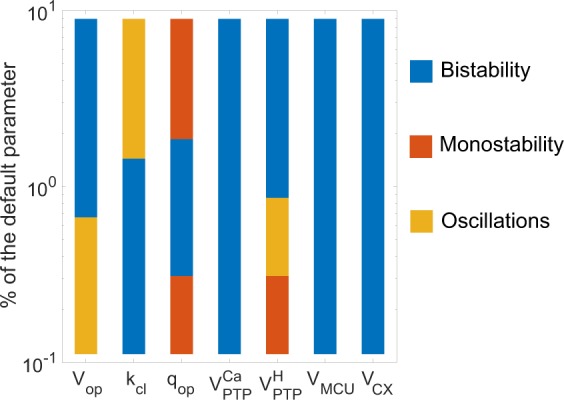
Range of bistability in the mPTP opening model. In blue: the system is bistable; in orange-coloured: the system has one stable steady-state; in yellow: the system oscillates at high $PTP$ values. Sensitivity analysis was performed by individually varying seven different parameters from 10 to 1000 % of their default values. See Fig. S3 for bistability conditions and notes on oscillatory behaviour.

To be biologically relevant, bistability in mPTP dynamics must be robust with respect to cell-to-cell variations. It is thus important to check that the bistable behaviour predicted by the mathematical model occurs for a large range of values of the kinetic parameters. We first analysed the extent of the domain of bistability when changing the values of the parameters of the model (Fig. 5, in blue). It is visible that bistability occurs in a quite extended range of values of all parameters. The existence of bistability is sensitive to the kinetic parameters that control the opening of the pore (${V}_{op}$ and ${k}_{cl}$). If the pore opens too slowly, or closes too fast, bistability is lost. Bistability is thus favoured by a given ratio between opening and closure rates (Fig. S3B). The most sensitive parameter is ${q}_{op}$, that regulates the threshold of inhibition of the pore by $\Delta \Psi$ (Fig. S3C). This parameter also alters the threshold of mPTP opening by Ca${}^{2+}$. Consequently, increasing this parameter shifts the whole bifurcation curve (Fig. 4A for example) to the left, until the bistable domain disappears from positive Ca${}^{2+}$ concentrations. On the opposite, decreasing the parameter shifts the bistable area to the right. For high ${q}_{op}$ values, the system is thus bistable, but we consider this situation as biologically not relevant as it requires the addition of an unrealistically large amount of Ca${}^{2+}$. Interestingly, the existence of bistability is extremely robust with respect to changes in the parameters related to Ca${}^{2+}$ fluxes (${V}_{MCU}$, ${V}_{CX}$ and ${V}_{PTP}^{Ca}$). Changing these parameters mainly impacts on the rates at which Ca${}^{2+}$ is exchanged between mitochondria and the medium. This relative insensitivity confirms that the relationship between $\Delta \Psi$ and $PTP$ is the core mechanism leading to bistability, whereas Ca${}^{2+}$ controls the transition between the two states. Finally, if the ion flux through the mPTP (${V}_{PTP}^{H}$) is weak, bistability is also lost (Fig. S3D). Indeed, the loss of $\Delta \Psi$ induced by mPTP opening is then much reduced, which weakens the positive feedback loop that generates bistability.

### Simulation of transient openings of the mPTP

The level of opening of the mPTP in its low conductance mode is in average very small. The associated Ca${}^{2+}$ fluxes are thus expected to be minimal. In intact cells, openings in this mode indeed take the form of short-lived, stochastic events as reported for permeabilised heart cells^29^ and astrocytes^30^. These random openings are associated with small-amplitude drops in $\Delta \Psi$ and mitochondrial Ca${}^{2+}$ and are localised to small areas of the cell.

**Figure 6:**
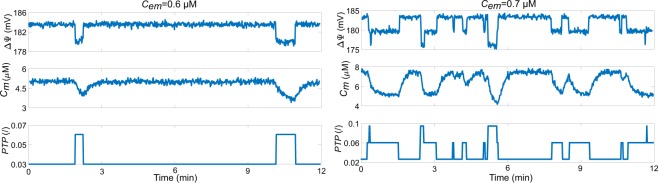
Stochastic opening of the mPTP. Time series of the evolution of $\Delta \Psi$, ${C}_{m}$ and $PTP$ for different ${C}_{em}$, obtained by simulations of a stochastic version of the model. The simulated system corresponds to the volume of a single mitochondrion exposed to a constant ${C}_{em}$.

We developed a stochastic version of the model to simulate transient mPTP openings (SI Appendix). As these openings remain highly localised, we simulated a small volume containing a single mitochondrion, and considered that the concentration of cytosolic Ca${}^{2+}$ around this mitochondrion (${C}_{em}$) remains constant. An increase in ${C}_{em}$ simulates an efflux from the ER, triggered by an external stimulus. At low ${C}_{em}$, simulations predict a noisy but stationary state for $\Delta \Psi$ and ${C}_{m}$, associated with a mPTP that is nearly always closed. If we increase ${C}_{em}$, small and rapid drops in $\Delta \Psi$, accompanied by some Ca${}^{2+}$ efflux from the mitochondrion, can be observed (Fig. 6). These isolated events correspond to sudden, random and brief openings of the pore in its low conductance mode. For higher Ca${}^{2+}$ concentrations, the number of events increases, as well as the mPTP open probability (Fig. S4). If the number of events becomes too large, changes in $\Delta \Psi$ and ${C}_{m}$ are quite irregular, as observed in heart mitochondria in suspension^31^.

## Discussion

We have provided a minimal description of the dynamics of the mPTP, in which we only considered the regulation of the pore by its main regulators, Ca${}^{2+}$ and $\Delta \Psi$. It is clear, however, that mPTP opening is inhibited by a variety of compounds, which include Mg${}^{2+}$, adenine nucleotides, cyclosporin A, mitochondrial H${}^{+}$, metabolic fluxes and environment, and stimulated by others, such as reactive oxygen species, organic phosphate or cyclophilin D^12,15,20,32^. Although the influence of most of these factors can partly be accounted for by their influence on $\Delta \Psi$ or Ca${}^{2+}$, a more accurate description of the mPTP regulation would be useful to account for some experimental observations. Inhibition of mPTP opening by protons is sometimes proposed as a primary mechanism of mPTP regulation^33^. In the present model, protons influence mPTP opening through membrane voltage, but not directly (see Eq. 4). It is known that the mPTP is open at pH $\ge$7.3-7.5 and closed at pH $<$7^34,35^. Because at rest, mitochondrial pH lies between 7.2 and 7.6^33,36^, and because an increase in [Ca${}^{2+}$]${}_{{\rm{m}}}$ leads to an increase in mitochondrial pH, direct H${}^{+}$ regulation of the mPTP cannot on its own account for the abrupt openings of the pore observed upon additions of Ca${}^{2+}$ (Fig. 3).

Focussing on the core regulatory mechanism of mPTP opening by Ca${}^{2+}$ and $\Delta \Psi$ allowed us to uncover the occurrence of bistability in an intermediate range of [Ca${}^{2+}$]${}_{{\rm{m}}}$. This bistability underlies the well-established existence of two operating modes displaying widely different average conductances. The proposed mechanism moreover agrees with the recent observation of a common molecular nature of the two modes of the pore, which is formed from the F${}_{{\rm{o}}}$F${}_{1}$ ATP synthase, at the interface between monomers within dimers^13^.

The bistable scenario proposed in this study contrasts with the description of the passage between the low- and the high-conductance mode of the pore as a switch controlled by a single threshold governed, for example, by mitochondrial pH^16,33,37,38^, [Ca${}^{2+}$]${}_{{\rm{e}}{\rm{m}}}$^39^, [Ca${}^{2+}$]${}_{{\rm{m}}}$^40^ or mitochondrial volume^41^. In Bazil *et al*.^15^, the switch is imposed by $\Delta \Psi$, and the value of the threshold is controlled by [Ca${}^{2+}$]${}_{{\rm{m}}}$, as in the present study. This $\sim$200-variable model is very detailed, as it includes regulation of the mPTP by a variety of factors as well as mitochondrial bioenergetics. It nicely reproduces experimental observations but is hardly usable for a bifurcation analysis. In a broader context, many processes governed by binary choices in cell biology have been described by bistability. This includes cell differentiation^42^, enzymatic reactions^43^, the cell cycle^26,44^, or bacterial communication^45^. One of the main advantages of bistability-controlled transitions is their robustness. In the case of the mPTP, this property plays a key role in relation to the very nature of the pore. Indeed, when the pore enters in a high conductance mode, it provokes a decrease in $\Delta \Psi$ and [Ca${}^{2+}$]${}_{{\rm{m}}}$. If a threshold-associated switch was at play, this decrease would bring the channel back in a low conductance mode, provoking in turn an increase in $\Delta \Psi$ and [Ca${}^{2+}$]${}_{{\rm{m}}}$, hence the opening of the channel again. These unrealistic back and forths between a fully- and a partially-open pore do not occur in a bistable scenario, which ensures that, once initiated, the full opening of the channel is maintained up to a point where largely depolarised mitochondria lead to cell death.

Much remains to be done to assess the physiological relevance of the low conductance mode of the pore in intact cells. We have touched this question by performing stochastic simulations of the model in conditions corresponding to a single mitochondrion facing different levels of Ca${}^{2+}$ in its surrounding cytosol. A stochastic approach is necessary because openings are transient and weak. The low level of mPTP opening computed in the deterministic bifurcation diagram (Fig. 4A) corresponds to an average over extended periods of time, during which the mPTP undergoes random, discrete openings (Fig. 6). In cells, such transient openings are thought to play an important role in releasing matrix Ca${}^{2+}$ to maintain mitochondrial homeostasis^46^. *In vivo*, mitochondria are morphologically and functionally heterogenous^47^, which can explain the large variety of changes in [Ca${}^{2+}$]${}_{{\rm{m}}}$ that have been reported experimentally^22^. Moroever, mitochondria are permanently rebuilt through fusion and fission that are promoted by mitochondrial movements^48^. The last two factors are expected to further increase the variability in mPTP openings, in addition to their inherent stochastic character, which again calls for a relatively noise-insensitive mode of switching between the two conductance modes.

The mPTP is involved in the pathophysiology of many diseases, ranging form ischemia/reperfusion injury to neurodegenerative disorders^49^. It would be useful to integrate the present description of the mPTP as a bistable molecular switch in comprehensive models of mitochondrial Ca${}^{2+}$ and metabolism^15,50,51^ to provide a detailed computational support to the experimental investigation of such pathologies^52^.

## Supplementary information

Supplementary Information.
