# Supplementary material for: Dual dynamics of mitochondrial permeability transition pore opening
Source: Sci Rep. 2020 Mar 3;10:3924. doi: 10.1038/s41598-020-60177-1 (PMC7054270; doi:10.1038/s41598-020-60177-1)
Supplement: Supplementary file 1 — Supplementary Information. [file 41598_2020_60177_MOESM1_ESM.pdf]

Dual dynamics of mitochondrial permeability transition pore opening

Benjamin Wacquier[1], **Laurent Combettes**[2], **and Geneviève Dupont**[1*]

[1]Unit of Theoretical Chronobiology, Faculté des Sciences, Université Libre de Bruxelles (ULB) CP231, B1050, Brussels, Belgium
[2]UMR-S 1174, Université Paris-Sud, 91405 Orsay, France
[*]gdupont@ulb.ac.be

Supplementary Data

Supplementary Text

Deterministic model

Our model combines an original description of the mPTP opening (Eqs. 1-7) and the model from Wacquier *et al.*, 2017[1], which has been developed to study Ca^{2+} dynamics in a mitochondrial suspension. The main differences with the model for intact cells[2] stands in 1) the absence of terms describing Ca^{2+} exchanges with the endoplasmic reticulum (i.e. Ca^{2+} fluxes through SERCA pumps and IP_3 receptors) as the ER is not present in a mitochondrial suspension; 2) the absence of adenine nucleotides description as the amounts of cytosolic ATP and ADP in the medium used in the experiments are very low. Thus, the evolution equations for cytosolic and mitochondrial ADP and ATP are not considered, and the associated fluxes are set to zero in the evolution equation for voltage[3,4]. Because there is no entry of ADP inside mitochondria, the concentration of mitochondrial ADP is also set to zero while $[ATP]_{mito}$ is maximal, i.e. the total concentration of mitochondrial adenine nucleotides.

The detailed kinetic expressions of the fluxes appearing in the evolution equations of the model are given by Eqs. 2-3 and S1-S6. Parameter values are listed in Table S1. The system of equations has been numerically simulated using the software XPPAUT[5] or MATLAB r2017b (ode23tb solver). The bifurcation diagrams have been drawn with these two softwares, either using the XPPAUT interface with AUTO, or numerically when using MATLAB.

- $$J_{in} = A(heav(t-t^*)heav(t^*+1-t)) \tag{S1}$$

- $$J_{MCU} = V_{MCU} \frac{\frac{C_{em}}{K_1}\left(1+\frac{C_{em}}{K_1}\right)^3}{\left(1+\frac{C_{em}}{K_1}\right)^4 + \frac{L}{\left(1+\frac{C_{em}}{K_2}\right)^{2.8}}} e^{p_1 \Delta\Psi} \tag{S2}$$

- $$J_{CX} = V_{CX}\left(\frac{C_m}{C_{em}}\right)\frac{e^{0.5FRT(\Delta\Psi - p_2)}}{(1+K_{CX}/C_m)} \tag{S3}$$

- $$J_{PDH} = k_{GLY}\frac{1}{q_1+\frac{[NADH]}{[NAD^+]}}\frac{C_m}{q_2+C_m} \tag{S4}$$

- $$J_O = k_o\frac{[NADH]}{q_3+[NADH]}\left(1+e^{\frac{\Delta\Psi - q_4}{q_5}}\right)^{-1} \tag{S5}$$

- $$J_{H,leak} = q_9\Delta\Psi + q_{10} \tag{S6}$$

Stochastic model

In the last section of the manuscript, we simulated a stochastic version of our deterministic model using a Gillespie's algorithm[6]. This approach allows to consider the effects of fluctuations and molecular noise that may be significant in individual mitochondria. This method requires that the variables and parameters expressed in concentration are converted into numbers of ions or molecules. A parameter, denoted Ω modulates these numbers of chemical species. It is directly proportional to the volume of the system. The iterative algorithm associates a propensity to each reaction or transport step in the model. Propensities depend on the numbers of molecules and kinetic parameters. At a given time, the algorithm randomly determines which reaction takes place. The probability of occurrence of a transition increases with its propensity. Then, the system is updated with respect to the chosen reaction, the time is incremented by a random number drawn from an exponential distribution function with mean equal to the inverse of the sum of the propensities, and the iterative process starts over.

In the stochastic section of this work, we simulated a single mitochondrion, and we used $\Omega = 5000$, which corresponds to a mitochondrial volume equal to 1 fL. The algorithm was applied to the Eqs. 1,4,6 and 7. We chose to maintain C_{em} constant, and thus did not consider the Eq. 5 in the algorithm.

Materials

Dulbecco's modified Eagle's medium and Williams' medium were from Life Technology (Invitrogen, Saint Aubin, France), Collagenase A from Boehringer (Roche Diagnostics, Meylan, France). Other chemicals were purchased from Sigma (Sigma-Genosys, Sigma-Aldrich Chimie, L'Isle d'AbeauChesnes, France).

Isolation of mitochondria from mouse liver

Mice livers were washed by retrograde perfusion in situ with a Sucrose Hepes buffer (250 and 20 mM, respectively) at pH 7.4. Livers were harvested and homogenised with an Ultra-Turrax (2000 rpm, 10 strokes), in the same medium at 4°C supplemented with 1 mM EGTA, 1 mM DTT, and a cocktail of Protease inhibitors (Invitrogen). The homogenate was centrifuged 5 min at 1500 x g. The supernatant was then centrifuged 10 min at 8000 x g to obtain the mitochondrial fraction. The mitochondrial fraction was washed one time in the measurement medium (Sucrose 50 mM, Succinate 10 mM, Tris 10 mM, CP 5 mM, pH 7.2 at room temperature (RT)), and resuspended in the same medium. Mitochondrial proteins concentration was determined with the BIORAD DC protein assay. 2 ml of mitochondrial suspension (1 mg/ml proteins) were transferred into the cuvette, a Varian Cary Eclipse Spectro fluorimeter, at RT and under continuous magnetic agitation.

Fluorescence measurements

Ca^{2+} measurements were performed with Fluo-4 (2.5 μM, $\lambda_{ex} = 480$, $\lambda_{em} = 520nm$). In some experiments, mitochondrial membrane potential ($\Delta\Psi$) was evaluated at the same time as the Ca^{2+} variations, using TMRM (invitrogen). We used TMRM in the quenching mode as $\Delta\Psi$ variations were expected to be fast[7]. In this case, 700 nM TMRM was added to the mitochondria 10 min before the start of the experiment. Then, the mitochondrial suspension was transferred into the cuvette, and Fluo-4 (2.5 μM) was added. The Varian Cary Eclipse Spectro fluorimeter allows us to record almost simultaneously the variations of extra-mitochondrial Ca^{2+} ($\lambda_{ex} = 480$, $\lambda_{em} = 520nm$) and $\Delta\Psi$ ($\lambda_{ex} = 550$, $\lambda_{em} = 580nm$). Note that, in the quenching mode, a mitochondrial depolarisation will result in increasing fluorescent signal[7].

Supplementary Tables and Figures

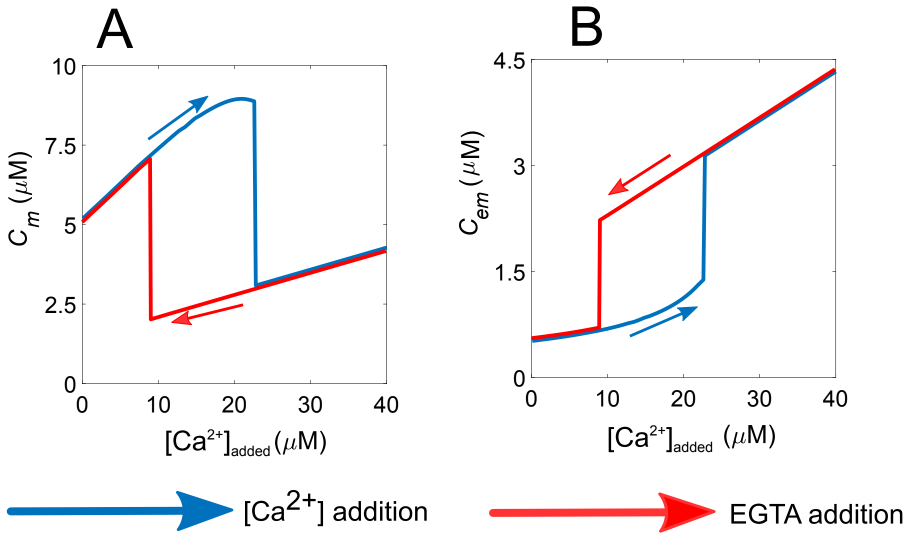

Figure S1. mPTP opening corresponds to a bistable switch. (**A-B**) Numerical bifurcation diagrams of C_m (**A**) and C_{em} (**B**) as a function of added Ca^{2+}. Blue curves stand for trajectories following Ca^{2+} additions (from left to right) whereas the red curves represent the trajectories following the Ca^{2+} removal (from right to left).

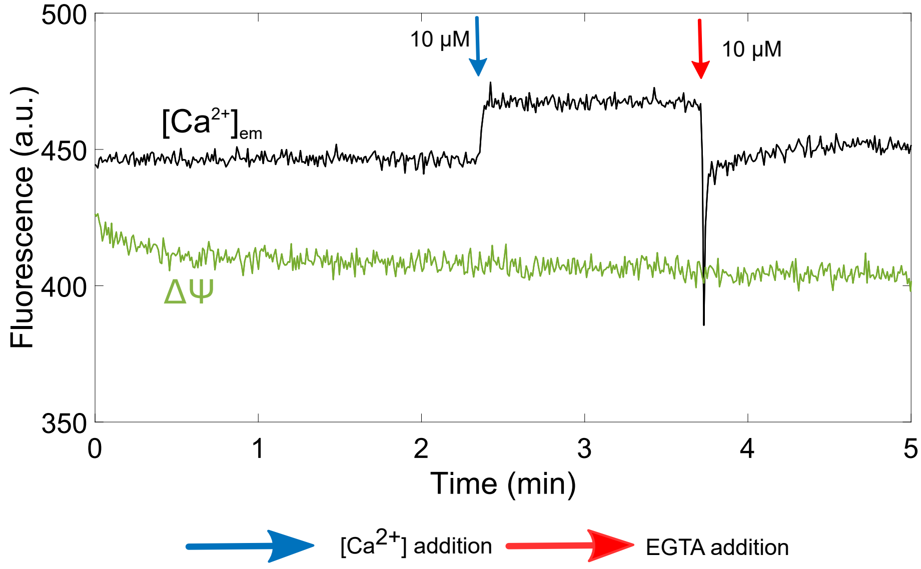

Figure S2. EGTA buffers Ca^{2+} additions in a 1/1 ratio. In a medium devoid of mitochondria, the addition of 10 μM EGTA buffers the 10 μM Ca^{2+} previously added to the medium (black line). This effect occurs on a time scale of ~10 s. The green line stands for TMRM fluorescence and shows that this ΔΨ indicator is not affected by the addition of EGTA.

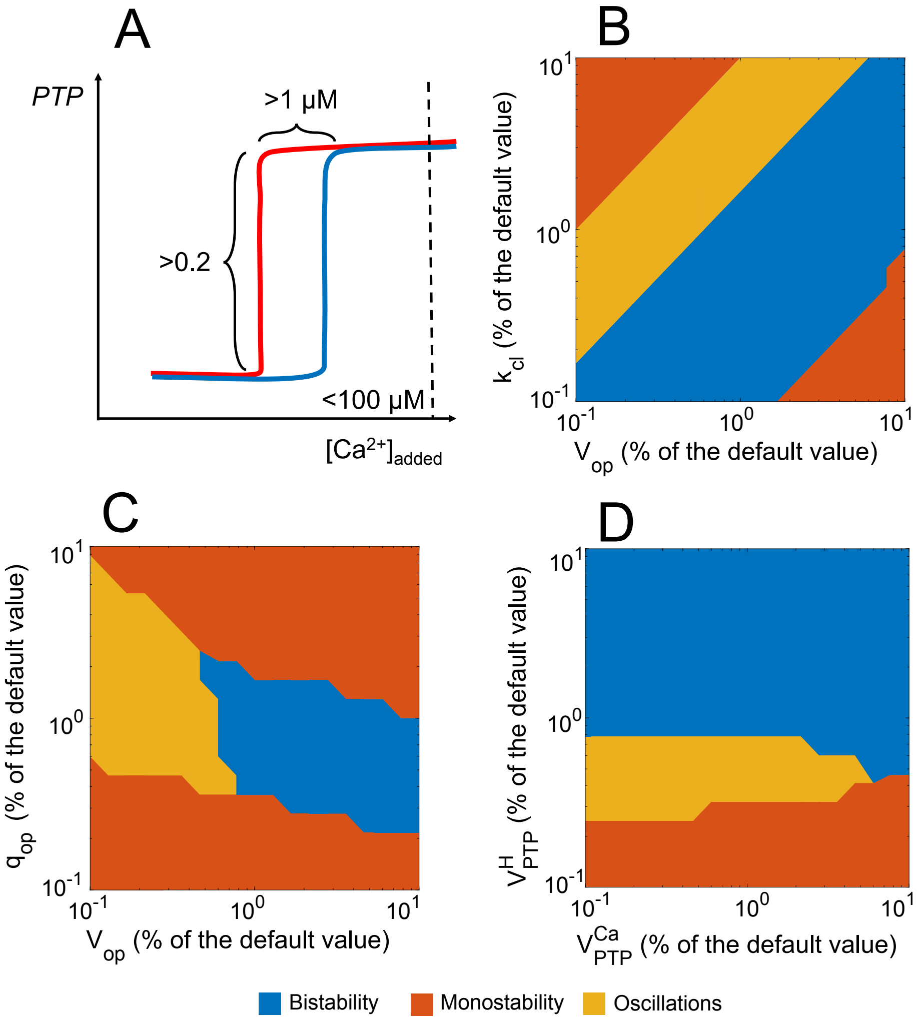

Figure S3. Robustness analysis of the mPTP opening model. (A) A set of parameter values was considered to give bistability when three conditions are fulfilled. We considered bistability to be meaningful from a biological point of view if its range of occurrence exceeds 1 μM of added Ca^{2+}, and if the two stable steady-states differ by at least 0.2 in terms of open mPTP ratio. As a last condition, we imposed that the state corresponding to the high conductance mode must be reached by the addition of less than 100 μM Ca^{2+}. (**B-D**) Two parameters space. The parameters are varied from 10 to 1000 % of their default values. The default values are those listed in the Table S1. In blue: the system is bistable; in orange-coloured: the system has one stable steady-state; in yellow: the system oscillates. These oscillations, occurring at high mPTP opening and with periods of a few hundreds of seconds, would correspond to a situation where cells are dying, as for a sustained mPTP opening in the high conductance mode.

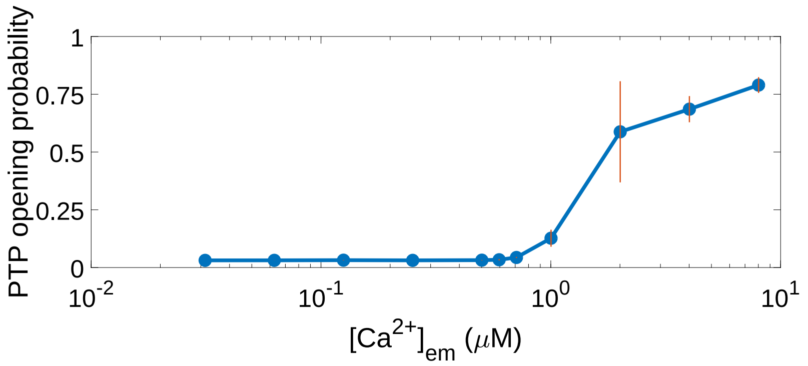

Figure S4. mPTP opening probability as a function of the Ca^{2+} concentrations in the medium. Each point is the average of seven 1000 s long independent simulations at a given concentration of extra-mitochondrial Ca^{2+}. Standard deviations are also indicated (in red). For C_{em} values lower than 1 μM, standard deviations are vanishingly small.

Table S1. List of parameter values.

*: these values depend on the experimental protocol. Parameter values are taken from the two-variable model for the dynamics of the mPTP (these parameter values were validated against experiments, see main text) and from our previously published model describing Ca^{2+} handling in mitochondrial suspensions.[1]

Parameter	Definition	Value	Ref.
a_1	Scaling factor between NADH consumption and variation of ΔΨ	20	2
C_p	Mitochondrial inner membrane capacitance divided by the Faraday constant	1.8 μ M.mV^{-1}	8
δ	Volumic ratio between mitochondria and the extra-mitochondrial medium	0.001*	1
F	Faraday constant	96480 C.mol^{-1}	
f_{em}	Fraction of free over buffer-bound Ca^{2+} in the extra-mitochondrial medium*	0.09*	1
f_m	Fraction of free over buffer-bound Ca^{2+} in hepatocyte mitochondria	0.0003	9
K_1	Dissociation constant for Ca^{2+} translocation by the MCU	25 μM	1
K_2	Dissociaion constant for MCU activation by Ca^{2+}	1.6 μM	1
k_{cl}	Rate constant for mPTP closure	0.02 s^{-1}	This work
K_{CX}	Dissociation constant for CX activation by mitochondrial Ca^{2+}	0.375 μM	10
k_{GLY}	Velocity of glycolysis (empirical)	450 μM.s^{-1}	8
k_o	Rate constant of NADH oxidation by ETC	600 μM.s^{-1}	8
L	Allosteric equilibrium constant for uniporter conformations	50	11
$[NAD]_m^{tot}$	Total concentration of mitochondrial nicotinamide adenine dinucleotides	250 μM	2
p_1	Voltage dependence coefficient of the MCU activity	0.1 mV^{-1}	2
p_2	Voltage dependence coefficient of the CX activity	91 mV	10
q_1	Michaelis-Mentens-like constant for NAD^+ consumption by Krebs cycle	1	8
q_2	$S_{0.5}$ value for activation of the Krebs cycle by Ca^{2+}	0.1 μM	2
q_3	Michaelis-Menten constant for NADH consumption by ETC	100 μM	8
q_4	Voltage dependence coefficient 1 of ETC activity	177 mV	8
q_5	Voltage dependence coefficient 2 of ETC activity	5 mV	8
q_9	Voltage dependence of the proton leak	2 μM.s^{-1}.mV^{-1}	8
q_{10}	Rate constant of the voltage-independent proton leak	-30 μM.s^{-1}	8
q_{11}	Voltage dependence coefficient of PTP opening	20 mV	This work
q_{12}	Voltage dependence coefficient 1 of ion fluxes through mPTP	50 mV	This work
q_{13}	Voltage dependence coefficient 2 of ion fluxes through mPTP	100 mV	This work
q_{op}	Voltage and mitochondrial Ca^{2+} dependence coefficient of PTP opening	12 mV.μM^{-1}	This work
R	Perfect gas constant	8315 mJ.mol^{-1}.K^{-1}	
T	Temperature	310.16 K	
V_{CX}	Rate constant of the Ca^{2+} exchangers (NCLX and HCX)	1.37 μM.s^{-1}	10
V_{MCU}	Rate constant of the MCU	6.10^{-4} μM.s^{-1}	1
V_{op}	Rate constant of mPTP opening	0.07 s^{-1}	This work
V_{PTP}^{Ca}	Rate constant of Ca^{2+} flux through mPTP	50 μM.s^{-1}	This work
V_{PTP}^{H}	Rate constant of protons flux through mPTP	45000 μM.s^{-1}	This work

References

1. Wacquier, B., Romero Campos, H. E., González-Vélez, V., Combettes, L. & Dupont, G. Mitochondrial Ca^{2+} Dynamics in Cells and Suspensions. *The FEBS J.* **284**, 4128–4142, DOI: 10.1111/febs.14296 (2017).

2. Wacquier, B., Combettes, L., Tran Van Nhieu, G. & Dupont, G. Interplay Between Intracellular Ca^{2+} Oscillations and Ca^{2+}-stimulated Mitochondrial Metabolism. *Sci. Reports* **6**, DOI: 10.1038/srep19316 (2016).

3. Chinopoulos, C. *et al.* A Novel Kinetic Assay of Mitochondrial ATP-ADP Exchange Rate Mediated by the ANT. *Biophys. J.* **96**, 2490–2504, DOI: 10.1016/j.bpj.2008.12.3915 (2009).

4. Kawamata, H., Starkov, A. A., Manfredi, G. & Chinopoulos, C. A Kinetic Assay of Mitochondrial ADP-ATP Exchange Rate in Permeabilized Cells. *Anal. Biochem.* **407**, 52–57, DOI: 10.1016/j.ab.2010.07.031 (2010).

5. Ermentrout, B. *Simulating, Analyzing, and Animating Dynamical Systems: A Guide to XPPAUT for Researchers and Students* (Society for Industrial and Applied Mathematics, 2002).

6. Gillespie, D. T. A General Method for Numerically Simulating the Stochastic Time Evolution of Coupled Chemical Reactions. *J. computational physics* **22**, 403–434 (1976).

7. Perry, S. W., Norman, J. P., Barbieri, J., Brown, E. B. & Gelbard, H. A. Mitochondrial Membrane Potential Probes and the Proton Gradient: a Practical Usage Guide. *BioTechniques* **50**, 98–115, DOI: 10.2144/000113610 (2011).

8. Bertram, R., Gram Pedersen, M., Luciani, D. S. & Sherman, A. A Simplified Model for Mitochondrial ATP Production. *J. Theor. Biol.* **243**, 575–586, DOI: 10.1016/j.jtbi.2006.07.019 (2006).

9. Fall, C. P. & Keizer, J. E. Mitochondrial Modulation of Intracellular Ca^{2+} Signaling. *J. Theor. Biol.* **210**, 151–165, DOI: 10.1006/jtbi.2000.2292 (2001).

10. Cortassa, S., Aon, M., Marbán, E., Winslow, R. L. & O'Rourke, B. An Integrated Model of Cardiac Mitochondrial Energy Metabolism and Calcium Dynamics. *Biophys. J.* **84**, 2734–2755, DOI: 10.1016/S0006-3495(03)75079-6 (2003).

11. Magnus, G. & Keizer, J. Model of Beta-cell Mitochondrial Calcium Handling and Electrical Activity. II. Mitochondrial Variables. *Am. J. Physiol. Physiol.* **274**, C1174–C1184, DOI: 10.1152/ajpcell.1998.274.4.C1174 (1998).
